# Serum-free production of anti-huCD20(hγ1)-IL2no-alpha immunocytokine: a promising therapeutic for B-NHL

**DOI:** 10.3389/fbioe.2026.1773646

**Published:** 2026-04-15

**Authors:** Debbie Laura Bergantiños Albernas, Ernesto Azahares Valdivia, Carlos Lázaro Pérez Rosales, Ana Laura Hurtado de Mendoza Arrieta, Claudia de las Mercedes Castellanos Díaz, Miguel Ángel González, Katya Sosa Aguiar, Tania Gómez Peña, Julio Felipe Santo Tomás Pompa, Julio Palacios-Oliva, Katya Rashida De la Luz Hernández, Tays Hernández, Ana Victoria Casadesús Pazos

**Affiliations:** 1 Immunobiology Direction, Center of Molecular Immunology, Havana, Cuba; 2 Process Development Direction, Center of Molecular Immunology, Havana, Cuba; 3 Department of Hematology, Hermanos Ameijeiras Hospital, Havana, Cuba; 4 Department of Animal Facilities, Center of Molecular Immunology, Havana, Cuba; 5 Quality Control Division, Center of Molecular Immunology, Havana, Cuba; 6 Commercial Management, Center of Molecular Immunology, Havana, Cuba; 7 Clinical Direction, Center of Molecular Immunology, Havana, Cuba

**Keywords:** anti-CD20, Chinese hamster ovary cells, IL2 mutein, immunocytokine, lymphoma, mTOR signaling, pseudo-perfusion

## Abstract

**Introduction:**

Mammalian cell cultures are widely used for producing complex biopharmaceuticals that require human-like post-translational modifications, such as antibody-based therapeutics. Traditionally, serum-supplemented media support high cell viability and productivity; however, regulatory and scientific requirements demand serum-free conditions for clinical-grade manufacture. Recently, a novel fusion protein, the anti-huCD20(hγ1)-IL2no-alpha immunocytokine (IC), was presented as a promising therapeutic alternative, mostly for relapsed or refractory (r/r) B-cell non-Hodgkin lymphoma (B-NHL) patients, considering currently approved therapies.

**Methods:**

Three Chinese hamster ovary clones (K1 strain) producing the anti-huCD20(hγ1)-IL2no-alpha IC were generated and adapted to serum-free suspension culture. We performed a kinetic characterization of one clone in two culture media with different nutritional compositions, evaluating cell growth, productivity, cell cycle progression and mTOR signaling. The IC was purified by Protein A, then evaluated for identity, aggregation profile, CD20 recognition, CTLL-2 cytokine activity, ex vivo B-cell depletion in PBMC from r/r B-NHL patients and antitumor efficacy in immunocompetent C57BL/6 mice bearing EL4-hCD20- cells.

**Results:**

The results demonstrated noticeable differences in cell growth and productivity in both batch and pseudo-perfusion performance, likely due to an influence on cell-cycle progression and mTOR signaling. The purified IC maintained its structural integrity while exhibiting an improved aggregation profile compared to serum-containing cultures. Furthermore, key biological activities, including B-cell depletion and antitumoral effects, remained intact.

**Discussion:**

This research highlights the successful serum-free production of a functional anti-huCD20(hγ1)-IL2no-alpha IC, reinforcing its potential for biopharmaceutical development.

## Introduction

Biological therapies have recently gained increasing relevance in cancer treatment, particularly in comparison with traditional drugs, and protein-based therapies constitute the majority of biological products that have received approval (Ebrahimi and Samanta, 2023). In contrast to chemical synthesis processes, the production of these therapies is complex due to the necessity of using live cells for their generation (O'Flaherty et al., 2020).

In 2022, Casadesus and colleagues at the Center of Molecular Immunology (CIM) developed the anti-huCD20(hγ1)-IL2no-alpha immunocytokine (IC), the first trifunctional IC to incorporate a mutated interleukin-2 (IL2no-alpha mutein) (Carmenate et al., 2013). This molecule is based on a human IgG1 version of rituximab (RTX), a chimeric monoclonal antibody (mAb) targeting human CD20 (Maloney et al., 1997). Although RTX, a clinically established agent, has significantly improved outcomes for patients with B-cell non-Hodgkin lymphoma (B-NHL), approximately one-third of treated individuals still experience relapse or are refractory (r/r) (Karsten et al., 2024; Bock and Epperla, 2025). Furthermore, despite advances in antibody–drug conjugates, CAR-T cell therapies, and bispecific T-cell engagers, limitations still persist, and r/r patients remain a significant clinical challenge (Dabkowska et al., 2024; Trabolsi et al., 2024).

The IL2no-alpha mutein was engineered with four point mutations (R38A, F42A, Y45A, and E62A) that abrogate interaction with the alpha chain of the receptor, thereby expanding natural killer (NK) and CD8^+^ T cells without promoting regulatory T cells, thus overcoming key limitations of native IL2 (Carmenate et al., 2013). The anti-huCD20(hγ1)-IL2no-alpha IC localizes the immunomodulatory effects of IL2no-alpha and the effector functions of RTX at the tumor site, enhancing the efficacy of both monotherapies. Preclinical models have demonstrated improved survival with doses of the IC that were ineffective for the parental molecules alone, highlighting its therapeutic potential as an alternative for RTX-resistant patients (Casadesus et al., 2022). Its safety profile and ability to combine targeted cytotoxicity with localized T-cell activation and NK cell engagement position it as an innovative approach that may reduce relapse rates observed in existing therapies (Casadesus et al., 2022; Trabolsi et al., 2024).

While initially produced in serum-supplemented media (SSM), stable clones capable of serum-free (SF) suspension growth are essential for scalable, clinical-grade manufacturing of this promising biopharmaceutical. Mammalian cell culture systems are labor-intensive and have lower productivity than other hosts; however, the quality of the proteins is significantly superior (Kim and Eberwine, 2010; Berlec and Strukelj, 2013). Chinese hamster ovary (CHO) cells are currently the most utilized host cells in the biopharmaceutical industry for producing antibody-based biological products (Xu et al., 2023; Yang et al., 2024). These cells ensure proper protein assembly and folding, as well as post-translational modifications similar to those found in human cells, high growth rates in serum-free media (SFM), high yield, and scalability (Knight et al., 2021; Ha et al., 2022; Zhang et al., 2022).

Currently, there has been a significant shift in certain biopharmaceutical manufacturing contexts from fed-batch to perfusion culture, motivated by an easier scaling-up process, superiority in volumetric productivity, maintenance of a steady high-quality environment (Bettinardi et al., 2020; Schulze et al., 2022; Liang et al., 2023), and higher purity and quality of the product (Walther et al., 2019). Optimizing the mammalian target of rapamycin (mTOR) activity and synchronizing cell-cycle progression have also been shown to enhance viable cell density (VCD) and product yield in CHO cell cultures through regulating cell growth, proliferation, and metabolism, thus making them critical parameters for scalable biopharmaceutical manufacturing (Ekim et al., 2011; Mao et al., 2024).

The mAb expression platform at the CIM, while initially based on NS0 host cells and electroporation (Dorvignit et al., 2012), has progressively shifted toward CHO cells, lentiviral vector transduction, SF chemically defined media, and suspension culture in perfusion mode to enhance scalability, productivity, and bioprocess robustness (Lao-Gonzalez et al., 2021; Boggiano-Ayo et al., 2023).

Here, we report the generation of stable CHO clones (K1 strain) (CHO-K1) adapted to SF suspension culture that produced the anti-huCD20(hγ1)-IL2no-alpha IC with adequate biophysical qualities. We also demonstrate, for one of these clones, that a pseudo-perfusion regimen and nutrient-rich medium support high-VCD, sustained productivity, and desirable biological properties of the produced IC through mTOR modulation. This study establishes a scalable and regulatory-compatible platform for the manufacture of engineered immunocytokines with potentially improved therapeutic efficacy.

## Materials and methods

### Anti-huCD20(hγ1)-IL2no-alpha plasmid construction

For anti-huCD20(hγ1)-IL2no-alpha expression, DNA coding for the light (LC) and heavy (HC) chain of the IC (Casadesus et al., 2022) were subcloned into the pCMX plasmid bearing the CMV promoter and an IgG signal peptide. The transcriptional cassettes encoding for LC and HC were next independently introduced into pL6WBlast (Center of Genetic Engineering and Biotechnology, Havana, Cuba) to obtain the lentiviral transfer vectors. The helper plasmids used for lentiviral transducing particle production were obtained from Invitrogen, United States.

### Cells, media, and cultures

Human embryonic kidney 293T cells (HEK-293T), Chinese hamster ovary K1 strain (CHO-K1) cells, Ramos (CD20^+^), and Jurkat (CD20^−^) were obtained from the American Type Culture Collection (ATCC). EL4-huCD20 cells (CD20^+^) were kindly provided by J. Golay (Ospedali Riunti di Bergamo, Bergamo, Italy). The murine T-cell line CTLL-2 was donated by Dr. A. Santos (Center of Genetic Engineering and Biotechnology).

HEK-293T cells were grown in DMEM/F12 medium (Gibco, USA) with 5% fetal bovine serum (FBS) (HyClone, GE HealthCare, USA) (DMEM/F12-FBS). Ramos, Jurkat, and CTLL-2 cell lines were cultured in RPMI medium (Gibco) with 10% FBS. EL4-huCD20 cells were cultured in IMDM medium (Gibco) with 20% FBS. DMEM/F12-FBS was used for CHO-K1 cell transduction at 2 μg mL^−1^ Blasticidin S HCl (Gibco) was used as selection agent. Transduced CHO-K1 cells were adapted to shaking conditions at 120 rpm (INFORS HT, Switzerland) in MB06 medium or in a 50:50 mixture of MB02 and PFHM II media (MB02-PFHM II) (Merck, Germany). All cells were incubated at 37 °C in a humidified 5% CO_2_ atmosphere. Cell count was performed using a Neubauer hemocytometer, based on the Trypan blue exclusion method (Strober, 2015).

MB02, PFHM II, and MB06 are protein-free media containing amino acids, vitamins, trace salts, glucose, and insulin in different proportions. MB02 contains protein hydrolysates whereas MB06 is chemically defined and supplemented with Cell Booster 5 (GE HealthCare, USA). Both formulations were additionally supplemented with 2 g L^−1^ sodium bicarbonate, 2.55 g L^−1^ HEPES, 1 g L^−1^ Pluronic® F-68 or Kolliphor® P188, 5 mg L^−1^ recombinant insulin, and 0.02 g L^−1^ ferric citrate.

### Immunocytokines, antibodies, and cytokines

The SFM ICs were affinity-purified from culture supernatants using a MabSelect SuRe Protein A column (GE HealthCare, Sweden). IC concentration was determined by measuring absorbance at 280 nm. No-alpha mutein was produced and purified at CIM as described in Carmenate et al. (2013). An isotope control IC, MOPC(hγ1)-IL2no-alpha, was also generated at CIM following an identical expression scheme to that of the SFM ICs and used as irrelevant IC (unpublished data). RTX mAb was purchased from Roche (anti-CD20; rituximab, MabThera-Roche, Türkiye).

### Quantification of IC expression levels by ELISA

The IC expression levels in cell culture supernatants were determined by sandwich ELISA. Microtiter 96-well plates (High Binding, Costar, USA) were coated with 3 μg mL^−1^ of goat anti-human IgG (γ-chain-specific) antibody (Sigma-Aldrich, USA) at 4 °C for 16 h. After washing the plates three times with washing buffer (phosphate buffered saline (PBS); Tween 20 at 0.05%, pH 7.5, PBS-T), the samples were diluted in blocking buffer (PBS-T containing bovine serum albumin (BSA) at 1% w/v) and then applied to the plates, which were incubated at 37 °C for 1 h. To detect the recombinant proteins, an HRP-conjugated goat anti-human kappa light chain antibody (Sigma-Aldrich) was used. To quantify the expression levels, a previously purified anti-huCD20(hγ1)-IL2no-alpha IC was used as a standard (standard curve range: from 7.8 to 500 ng mL^−1^). After every step of incubation, the plates were washed thrice with washing buffer PBS-T. Samples were analyzed in duplicate.

### Stable cell line generation

The recombinant lentiviral transducing particles were produced in HEK-293T cells and used to stably transfect CHO-K1 cells following Toledo et al. (2009) with modifications. After five rounds of transduction, CHO-K1 cells were seeded in 96-well plates in DMEM/F12 supplemented with 5% FBS and 2 μg mL^−1^ Blasticidin S HCl (selection medium) to generate oligoclones. The plates were incubated at 37 °C in a 5% CO_2_ atmosphere for 10 days. Oligoclones exhibiting the highest intracellular IC expression levels were subsequently expanded to 24-well plates. At this stage, cells were directly transferred from medium containing FBS to a chemically defined, protein-free medium (MB06) without a stepwise FBS reduction. Specifically, cells were transferred from 100 µL of DMEM/F12-FBS 5% to 0.5 mL of MB06 containing 2 μg mL^−1^ Blasticidin, resulting in an FBS concentration of 1%. Upon reaching confluence, the culture was split into two wells, each brought to a final volume of 1 mL with MB06/Blasticidin, yielding an FBS concentration of 0.25%. After approximately 4 days, cells were transferred to 25-cm^2^ T-flasks, the volume was adjusted to 7 mL with MB06/Blasticidin (residual FBS concentration of 0.07%), and cultures were subsequently maintained in MB06. T-flasks were incubated in a vertical position at 37 °C in a 5% CO_2_ atmosphere with orbital shaking.

To select the highest expressing oligoclones for subsequent cloning, cells were seeded at 0.5 × 10^6^ cells mL^−1^ in 10 mL of MB06 medium in 25-cm^2^ T-flasks in triplicate and incubated for 7 days under the same conditions. The selected oligoclones were cloned by limiting dilution at 0.5 cells per well in 96-well plates and incubated for 14 days. After each limiting dilution, FBS elimination was performed as previously described. Single-colony wells were screened by ELISA to identify top IC producers, and intracellular HC polypeptide content was assessed to evaluate cell population homogeneity. For this purpose, cells were fixed and permeabilized with Cytofix/Cytoperm (BD Pharmingen, USA), stained with FITC goat anti-human IgG (γ-chain-specific) antibody (Sigma-Aldrich), and analyzed on a Gallios flow cytometer (Beckman Coulter, USA). Data were processed using FlowJo vX 0.7 (Tree Star Inc., USA).

### Serum-free medium adaptation

Following confirmation by intracellular staining, selected clones were adapted to grow in SFM through successive passages in either MB02-PFHM II or MB06 media. Cells were seeded at a density of 0.5 × 10^6^ cells mL^−1^ in 125 mL Erlenmeyer flasks with a working volume of 30 mL and incubated at 37 °C in a 5% CO_2_ atmosphere with orbital shaking. Every 48 h, cells were counted using a hemocytometer and the trypan blue exclusion method; cell density was subsequently adjusted to 0.5 × 10^6^ cells mL^−1^, while viability and morphology were monitored. Cells were considered fully adapted after six to eight passages. Once a consistent doubling time of 48 h was achieved, viability remained above 98%, and no cell aggregates were observed in the culture.

### Batch culture in MB02-PFHM II and MB06 SFM

Cells were inoculated at 0.5 × 10^6^ cells mL^−1^ in 125 mL flat-bottom shake flasks (Corning, USA) containing a working volume of 50 mL. Cultures were maintained under controlled conditions at 37 °C, 5% CO_2_, and continuous shaking for 7 days, using MB02-PFHM II and MB06 SFM.

### Pseudo-perfusion culture in MB02-PFHM II and MB06 SFM

For pseudo-perfusion experiments, the same initial and culture conditions as those applied in batch culture were maintained. Medium exchange commenced once VCD reached 4 × 10^6^ cells mL^−1^ at a rate of 0.5 vvd (volume of fresh medium per volume of culture per day). When the VCD increased to 8 × 10^6^ cells mL^−1^, bleedings were initiated, and the medium exchange rate was subsequently raised to 0.7 vvd. From the first day of pseudo-perfusion, cell suspensions were centrifuged daily (1,000 g, 5 min, room temperature) to promote cell retention, followed by medium exchange. Specifically, once bleedings were initiated, 35 mL of culture was extracted every 24 h; of this, 0–10 mL was discarded, and the remaining volume was centrifuged at 1,000 *g* for 5 min. The resulting pellet was resuspended in 35 mL of fresh medium and reintroduced into the culture.

### Calculation of specific rates

Samples for cell counting and IC concentration analysis were collected from both batch and pseudo-perfusion cultures every 24 h. Cell density and viability were assessed using a hemocytometer and the trypan blue exclusion method, while IC expression was quantified by ELISA, as previously described. The trapezoidal method was applied to calculate the integral time of viable cell concentration (IVCC; ∫*Xvdt*; 10^8^ cells h mL^−1^). The maximum growth rate (μ_max_; h^−1^) and specific productivity (qp; pg·cell^−1^·day^−1^; pcd) were determined by plotting VCD or IC concentration, respectively, against IVCC. Values of μ_max_ and qp were derived from the slopes of these plots. The cell-specific perfusion rate (CSPR) was calculated daily, based on perfusion rates and mean VCD. All kinetic parameters were obtained from two biological replicates and are reported as mean ± standard error of the mean (SEM).

### Cell-cycle analysis

For cell-cycle analysis, 1 × 10^6^ cells were collected on days 6 and 16 from pseudo-perfusion in both media, washed with cold PBS with gentle shaking, fixed in 1 mL 70% molecular-grade ethanol for 30 min, and stored at 4 °C. Cells were then incubated in 50 μg mL^−1^ propidium iodide (Sigma-Aldrich) with 50 μL RNase A (Life Technologies, USA) in the dark for 15 min, placed on ice for 30 min, and kept in the dark until flow cytometry analysis.

### SDS-PAGE and Western blotting

Purified proteins were analyzed on 7.5% and 12% SDS-PAGE under non-reducing and reducing conditions, respectively, as described by Laemmli (1970). Cell culture supernatant samples (30 µL) or 5 µg of purified proteins were loaded. For Western blot analysis, proteins were transferred by electric field to PVDF membranes (Whatman, USA) using a wet transfer system (Bio-Rad, USA). After blocking for 1 h in TBS with 0.1% (v/v) Tween 20 (TBST) and 5% (w/v) BSA, HRP-conjugated goat anti-human kappa light chain antibody and HRP-conjugated goat anti-human IgG (γ-chain-specific) antibody (Sigma-Aldrich) were used for the detection of purified proteins. Mouse anti-IL2 polyclonal antibody (Center for Genetic Engineering and Biotechnology, Sancti Spiritus, Cuba) was used as a primary antibody before HRP-conjugated goat anti-mouse antibody (Bio-Rad) was added as a secondary revealing antibody. RTX and SSM IC were used as controls. In all cases, Broad Range molecular weight marker (Cell Signaling Technology) was used.

For mTOR, p-mTOR (S2448), P70S6K, p-P70S6K (S371), p-AMPK (T172), and β-actin detection cells from days 4 and 16 of pseudo-perfusion were lysed in protease inhibitor cocktail and cell lysis reagent (Sigma-Aldrich). The samples were vortexed for 1 min and then centrifuged at 900 *g* for 30 min at 4 °C. The supernatant was stored at −20 °C. After protein transfer and blocking, the membranes were incubated overnight with the respective rabbit primary antibodies (Cell Signaling Technology, USA) in TBST containing 2% (w/v) BSA. The secondary antibody, HRP-conjugated anti-rabbit IgG (Cell Signaling Technology), was then applied. In all cases, detection was carried out using an enhanced chemiluminescence (ECL) kit from Santa Cruz Biotechnology and imaged by ImageJ 2.1 (National Institutes of Health, USA).

### Size-exclusion chromatography and dynamic light scattering analysis

Size-exclusion high-performance liquid chromatography (SEC-HPLC) was performed in a TSKgel G3000SWXL (5 μm, 7.8 mm × 300 mm) column on an LC 2030C HPLC system from Shimadzu (Japan). In the same run sequence, thyroglobulin (670 kDa) and aldolase (158 kDa) were processed as analytical control from High Molecular Weight calibration standard (Cytiva, Sweden).

Particle size was determined by dynamic light scattering (DLS) using a DelsaNano C particle analyzer (Beckman Coulter, USA) equipped with a 658 nm, 30 mW laser. Measurements were performed in triplicate in glass cuvettes at a 165° scattering angle and 25 °C, with a 50 μm pinhole. Data were processed using the instrument’s software with the Cumulant and CONTIN models.

### Recognition of CD20-positive cells

For CD20 recognition on the cell surface, EL4-huCD20, Ramos, or Jurkat cells (3 × 10^5^) were incubated with the ICs or mAb on ice for 30 min and washed with PBS. The binding of antibodies or ICs was detected by incubation with FITC-conjugated goat anti-human IgG (Fc-specific) (Sigma-Aldrich) for 30 min on ice, followed by flow cytometry. Mean fluorescence intensity (MFI) was determined with FlowJo vX 0.7 software (Tree Star Inc.).

### Proliferation assays

Proliferation analyses were performed as per Casadesus et al. (2022) with minor modifications. Cells were treated with IL2no-alpha mutein, RTX, or ICs at different concentrations.

### B-cell depletion assay

The anti-CD20 mAb activity in the SFM IC was evaluated using a B-cell depletion assay. To address this, peripheral blood mononuclear cells (PBMC) were isolated by Ficoll-Paque™ PLUS density gradient centrifugation (GE HealthCare, Sweden) from three patients with r/r B-NHL who had previously received R-CHOP as first-line treatment—two with diffuse large B-cell lymphoma (DLBCL) and one with follicular lymphoma (FL). Fresh PBMC (3 × 10^5^ cells) were cultured with 18 nM of IC, RTX, or an irrelevant control IC for 96 h at 37 °C and 5% CO_2_. Cells were then collected, washed, stained with anti-CD45-PE (phycoerythrin, BD Invitrogen) and anti-CD19-PECy7 (phycoerythrin cyanine-7, BD Pharmingen), and analyzed by flow cytometry.

### Mice

Female C57Bl/6 mice, 5–8 weeks old and weighing 18–20 g, were obtained from the National Center for Laboratory Animal Breeding (Havana, Cuba). After a 7-day acclimation at the CIM animal facility, food and water were provided *ad libitum*. Experiments followed International Laboratory Animals Resources guidelines and CIM standardized protocols. All procedures were approved by the Institutional Animal Care and Use Committee and complied with ARRIVE Guidelines 2.0 (https://www.arriveguidelines.org/resources).

### EL4-huCD20 tumor model

C57Bl/6 mice were inoculated intravenously (IV) in the tail vein with 5 × 10^5^ EL4-huCD20 cells per mouse in 200 µL PBS on day 0. Afterward, they were randomized into the different treatment groups. RTX (150 µg/injection/mouse), SFM IC (20 µg/injection/mouse), or irrelevant IC (20 µg/injection/mouse) were administered intraperitoneally at days 1, 4, and 7 after tumor inoculation. Mice were euthanized when signs of disease appeared (e.g., prostration, paralysis, body weight drop). All non-commercial proteins were quality tested before the *in vivo* assay (purity, integrity, *in vitro* activity, and endotoxin levels).

### Statistical analysis

Statistical significance (p < 0.05) was determined by one-way ANOVA with Dunn’s *post hoc* test for multiple comparisons. To assess survival differences, Kaplan–Meier curves were produced and analyzed by log-rank tests. Statistical analysis was performed using GraphPad Prism version 7.0 software (GraphPad Software, Inc., USA).

## Results

### Obtaining IC-expressing CHO-K1 cell oligoclones and clones in SFM

**FIGURE 1:**
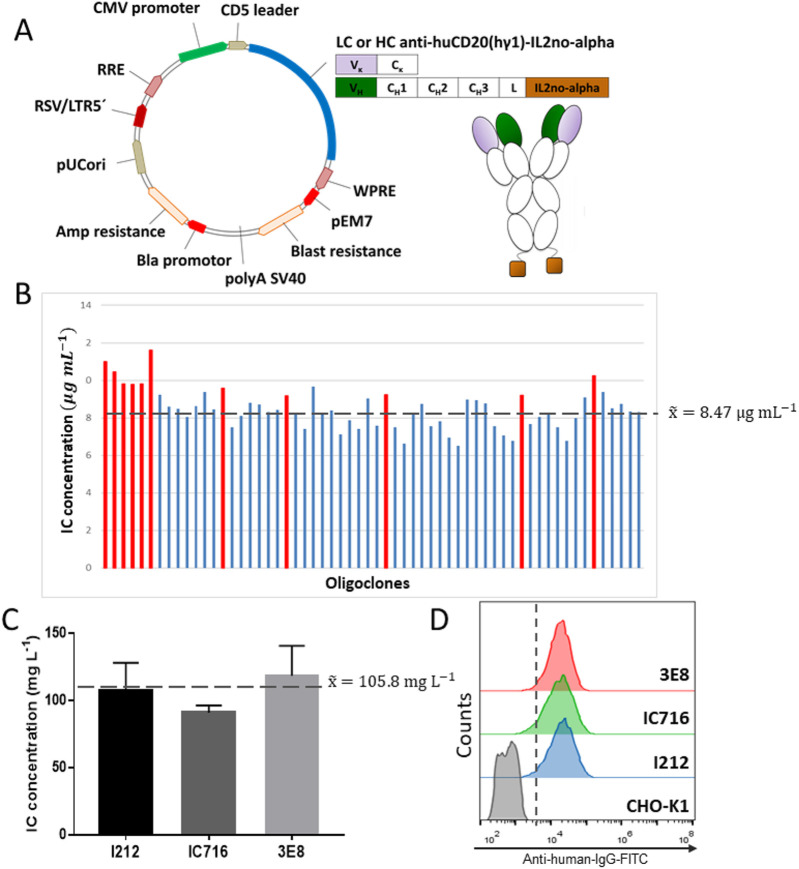
Expression and intracellular HC determination of CHO-K1 oligoclones producing anti-huCD20(hγ1)-IL2no-alpha under serum-free conditions. **(A)** Schematic representation of the lentiviral transfer plasmids used for anti-huCD20(hγ1)-IL2no-alpha expression and IC structure. pL6WBlast vector encoding the light (LC) or heavy (HC) chain of anti-huCD20(hγ1)-IL2no-alpha IC (pEM7, EM7 promoter; blast resistance, blasticidin resistance gene; polyASV40, SV40 polyadenylation signal; Bla promotor, β-lactamase (bla) promoter; Amp resistance, ampicillin resistance gene; pUCori, pUC origin of replication; RSV/LTR5’, Rous sarcoma virus 5’ long terminal repeat; RRE, Rev response element; CMV, cytomegalovirus promoter; CD5, leader CD5 signal peptide; WPRE, woodchuck hepatitis virus posttranscriptional regulatory element; V_k_ (purple), variable region of LC; C_k_, constant region of LC (kappa); VH (green), variable region of HC; CH, constant region of HC; L, amino-acid linker (Gly_4_Ser)_3_GT; IL2no-alpha mutein (brown)). **(B)** Evaluation of IC expression in CHO-K1 oligoclones using 96-well plate assay. Transduced cells were expanded to 96-well plates in selection medium. Plates were incubated at 37 °C and 5% CO_2_. IC concentration in cell culture supernatants was measured by ELISA after 14 days. To quantify expression levels, a standard curve of anti-huCD20(hγ1)-IL2no-alpha IC was made. The discontinuous line indicates the average (x^–^) IC level of the evaluated oligoclones. Red bars indicate the selected oligoclones for their expansion and SFM adaptation. **(C)** Expression levels of IC in three CHO-K1 clones cultured in SFM using T-flasks. Cells were seeded in 25-cm^2^ T-flasks in 10 mL of MB06 medium and incubated in vertical position and shaking conditions at 37 °C in 5% CO_2_. After 7 days, IC levels in culture supernatant were quantified by ELISA using the same standard IC curve. The data correspond to mean ± SEM. The discontinuous line indicates the average level of IC for the three selected clones. **(D)** Assessment of HC polypeptide content of cell clones by flow cytometry. Intracellular HC polypeptides of ethanol-fixed and permeabilized cells were measured using a FITC-labeled goat anti-human IgG (γ-chain-specific) antibody.

The anti-huCD20(hγ1)-IL2no-alpha was stably expressed in CHO-K1 cells following the strategy outlined in Supplementary Figure S1. A schematic overview of the lentiviral transfer plasmids employed for IC expression and the corresponding IC structure is presented in Figure 1A. Transduced cells were subcloned, and after 14 days, IC presence in supernatants was confirmed by ELISA. The best oligoclones (shown in red), with an average IC concentration of 8.47 μg mL^−1^, were selected for their expansion (Figure 1B). Oligoclones cultured for 3 weeks in MB06 SFM under shaking conditions showed >90% viability and consistent growth rate. IC production was assessed using the T-flask format over 7 days, as previously described, to select the best candidates to be used in further steps of limiting dilution cloning. Several steps of limiting dilution yielded clones I212, IC716, and 3E8. IC production of these clones averaged 105.6 mg L^−1^ in the T-flask format (Figure 1C), and intracellular HC staining confirmed population homogeneity (Figure 1D).

These clones were successfully adapted to suspension culture in SFM, exhibiting sustained growth throughout the process. Full adaptation was achieved after 16–20 total passages, as evidenced by appropriate morphology, viability exceeding 98%, and the absence of visible aggregates. The clones were additionally examined for *Mycoplasma* contamination and tested negative (data not shown). No protein degradation of the IC was detected during either the adaptation or production process.

### Purity and identity studies

**FIGURE 2:**
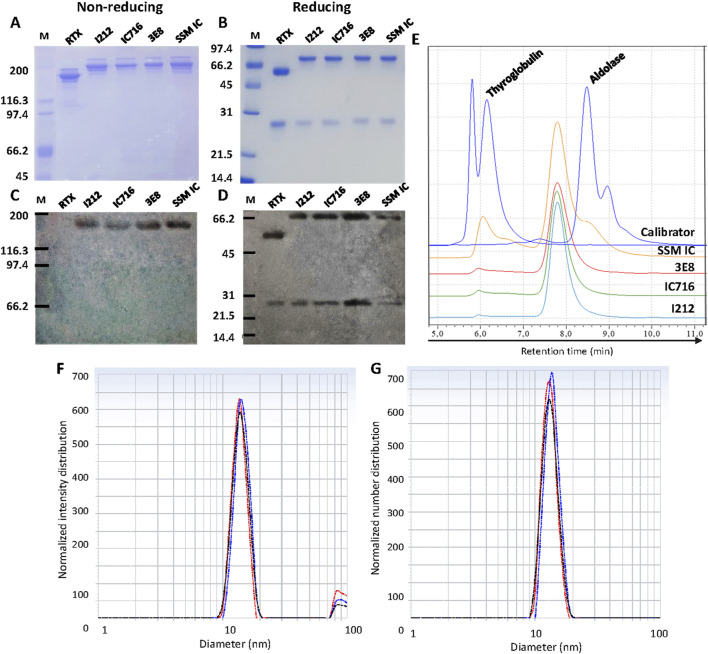
Characterization of SFM anti-huCD20(hγ1)-IL2no-alpha. SDS-PAGE of purified ICs under non-reducing **(A)** and reducing **(B)** conditions. Western blot analysis under non-reducing conditions using anti-IL2 mouse polyclonal antibody (produced and purified at CIM) and HRP-conjugated goat anti-mouse **(C)** and under reducing conditions, using HRP-conjugated anti-human kappa light chain and IgG (γ-chain-specific) antibodies **(D)**. M, broad range molecular weight marker. Gel was cropped to remove irrelevant lanes; brightness and contrast were adjusted uniformly across the entire image. **(E)** SEC-HPLC representative profiles of the SFM IC from the I212 (blue), IC716 (green), and 3E8 (red) CHO-K1 clones compared to SSM IC (orange). In dark blue are represented, from left to right, peak 1: aggregates, peak 2: thyroglobulin, and peak 3: aldolase from the calibrator. Normalized intensity distribution **(F)** and normalized volume distribution **(G)** graphs plotted against particle diameter (nm) of SFM IC evaluated by DLS. It represents three overlaid independent runs of the sample.

The three clones, fully adapted to SFM, were cultured in batches and yielded supernatants that were subsequently purified by Protein A chromatography. SDS-PAGE confirmed the presence of bands at approximately 180 kDa under non-reducing conditions (Figure 2A), consistent with the expected molecular mass of the full IC (150 kDa from the antibody backbone plus 15 kDa from each cytokine moiety). Under reducing conditions, two bands corresponding to the LC at 25 kDa and the HC at 65 kDa were observed (Figure 2B). This allowed the subsequent identification by Western blot of the cytokine moiety (Figure 2C) and the IC heavy and light chains (Figure 2D).

The chromatographic profile showed a main peak with an average retention time of 7.791 ± 0.031 min of a total of nine batches (three from each clone), consistent with a monomeric species (Figure 2E) and preceding the aldolase calibrator (158 kDa, 8.480 min). The monomeric fraction was >92% in all analyzed samples *versus* 77% for SSM IC. The process demonstrated high consistency (Supplementary Table S1). DLS analysis of the purified IC revealed a cumulative particle size of 16.07 ± 0.71 nm and a very low polydispersity index (PDI) of 0.018 ± 0.010, indicative of a monodisperse sample (Figures 2F,G).

### Batch and pseudo-perfusion culture in MB02-PFHM II and MB06 culture media

**FIGURE 3:**
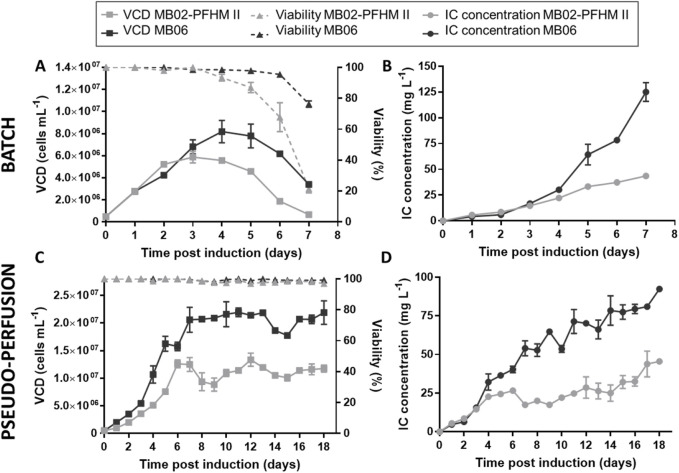
Kinetic analysis of CHO-K1 clone 3E8 cultured in batch and pseudo-perfusion modes using MB02-PFHM II and MB06 media. Growth profile and IC production of 3E8 cell clone in batch (**A,B**, respectively) and pseudo-perfusion (**C,D**, respectively) in MB02-PFHM II (light gray) and MB06 (dark gray). Cells were seeded in 125 mL shake flasks in 50 mL of medium and incubated at 37 °C with 5% CO_2_ and shaking conditions. Experiments were performed in duplicate. Every 24 h, cell culture samples were collected, and viable cell density (VCD), cell viability, and IC concentration in supernatant were determined.

Due to its superior performance, the IC-expressing CHO-K1 clone 3E8 was selected for subsequent kinetic studies. Among the three tested clones, 3E8 exhibited the highest IC production in 100 mL MB06 batch culture (Supplementary Figure S2) and demonstrated enhanced performance in a preliminary pseudo-perfusion culture in MB02-PFHM II (data not shown). Table 1 summarizes the productivity and growth characteristics of this clone in batch cultures using MB02-PFHM II and MB06 media. As shown in Figure 3A, maximal VCD reached 8.19 ± 1.01 × 10^6^ cells mL^−1^ in MB06, compared to 5.88 ± 0.53 × 10^6^ cells mL^−1^ in MB02-PFHM II. Cell viability remained above 80% after 5 days in both media; however, viability declined sharply in MB02-PFHM II between days 6 and 7. By day 6, viability in MB06 was still above 90%, whereas in MB02-PFHM II it had decreased to approximately 67%. The maximal IC concentration and IVCC were greater in MB06 (125.11 ± 9.19 mg L^−1^; 9.10 ± 0.16 × 10^8^ cells h mL^−1^) than in MB02-PFHM II (43.59 ± 1.02 mg L^−1^; 6.35 ± 0.09 × 10^8^ cells h mL^−1^) (Figure 3B). However, MB02-PFHM II showed a slightly higher μ_max_ than MB06 (0.033 ± 0.001 h^−1^ vs. 0.028 ± 0.000 h^−1^).

**TABLE 1:** Productivity and growth parameters of IC-expressing CHO-K1 clone 3E8 in batch and pseudo-perfusion culture using MB02-PFHM II and MB06 media.

Culture mode	Medium	VCD_max_ (10^6^ cells mL^−1^)	IVCC (10^8^ cells h mL^−1^)	μ_max_ (h^−1^)	[IC]_max_ (mg L^−1^)	qp (pcd)	Mean CSPR (nL cell^−1^ day^−1^)
Batch	MB02-PFHM II	5.88 ± 0.53	6.35 ± 0.09	0.033 ± 0.001	43.59 ± 1.02	1.37 ± 0.01	n.a
	MB06	8.19 ± 1.01	9.10 ± 0.16	0.028 ± 0.000	125.11 ± 9.19	2.40 ± 0.19	n.a
Pseudo-perfusion	MB02-PFHM II	13.31 ± 1.19	38.32 ± 1.08	0.019 ± 0.001	45.50 ± 1.04	1.92 ± 0.19	0.064 ± 0.006
	MB06	21.97 ± 0.80	69.89 ± 1.22	0.026 ± 0.003	92.36 ± 1.78	2.98 ± 0.07	0.036 ± 0.006

*VCD*_max_, maximum viable cell density; *IVCC*, time integral of viable cell concentration; *μ*_max_, maximum growth rate; *[IC]*_*max*_, maximum IC; concentration, *qp* specific productivity; *CSPR*, cell-specific perfusion rate; *n. a*. not applicable.

The next step was to evaluate the performance of the cell clone 3E8 at high cell density in pseudo-perfusion culture. Cells were cultured for 18 days, with an exponential phase of cell growth that lasted for 6–7 days. The maximum VCDs reached in this experiment were 13.31 ± 1.19 × 10^6^ cells mL^−1^ in MB02-PFHM II and 21.97 ± 0.80 × 10^6^ cells mL^−1^ in MB06. In both cases cell viability was above 97% during the entirety of the experiment (Figure 3C). Consistent with shaker flask batch cultures, the IC concentration in the supernatant was approximately two-fold higher in MB06 compared to MB02-PFHM II, with values of 92.36 ± 1.78 mg L^−1^ and 45.50 ± 1.04 mg L^−1^, respectively (Figure 3D). This enhancement of the product concentration observed in MB06 was associated with an increased qp (2.98 ± 0.07 pcd vs. 1.92 ± 0.19 pcd) and a lower mean CSPR during the stationary phase (0.036 ± 0.006 nL·cell^−1^·day^−1^ vs. 0.064 ± 0.006 nL·cell^−1^·day^−1^).

Supernatants were analyzed on days 4, 6, and 16 by non-reducing SDS-PAGE, showing a major band near 180 kDa (Supplementary Figure S3). Additionally, anti-huCD20(hγ1)-IL2no-alpha, purified from 3E8 pseudo-perfusion supernatants, exhibited characteristics similar to the other batches, as checked by SDS-PAGE (Supplementary Figure S3) and SEC-HPLC (Supplementary Figure S4).

### Nutrient availability influences cell cycle and mTOR signaling

**FIGURE 4:**
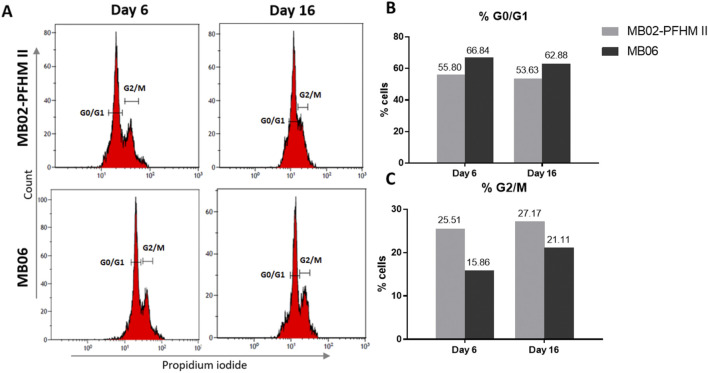
Cell-cycle distribution of recombinant CHO-K1 clone 3E8 on days 6 and 16 of pseudo-perfusion culture. Histograms **(A)** and graphical representation of cell percentage in G_0_/G_1_ **(B)** and G_2_/M **(C)** phases for each day on each culture medium are shown. Cells were stained with propidium iodide and analyzed for their DNA content by flow cytometry.

We also explored the effect of nutrient availability on cell cycle during pseudo-perfusion. Flow cytometry analysis was conducted on cells harvested on days 6 and 16 from both culture media. The cell-cycle assessment revealed that cells cultured in MB06 exhibited a higher percentage located in G_0_/G_1_ than in MB02-PFHM II and a lower percentage in the G_2_/M phase during the analyzed days (Figures 4A–C).

**FIGURE 5:**
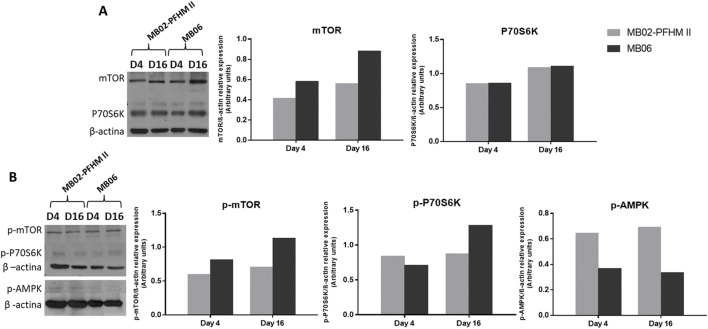
mTOR signaling regulation in CHO-K1 clone 3E8 cultured under pseudo-perfusion conditions. Equal number of cells from days 4 (D4) and 16 (D16) of pseudo-perfusion cultures of the 3E8 clone in MB02-PFHM II and MB06 media were lysed and analyzed. mTOR, p-mTOR, P70S6K, p-P70S6K, and p-AMPK (T172) were detected. β-actin was used to normalize for loading. Western blots (left) and graphical representations of relative expression levels for total **(A)** and phosphorylated proteins **(B)**. Films were cropped to remove irrelevant lanes; brightness and contrast were uniformly adjusted.

In addition, the mTOR signaling pathway was analyzed through Western blot on days 4 and 16, corresponding to the exponential growth and the stationary phases, respectively. In terms of mTOR levels, both the total and its active phosphorylated form (p-mTOR) were consistently higher in MB06 than in MB02-PFHM II at both time points (Figures 5A,B). Markedly, levels of total and phosphorylated mTOR remained quite stable over time in MB02-PFHM II while they exhibited an increase in MB06. Total P70S6K levels, a direct downstream effector of the mTOR complex 1 (mTORC1) pathway, were similar in both culture media on both days. Conversely, its phosphorylated form (p-P70S6K) showed a different trend: it was slightly higher in MB02-PFHM II than in MB06 on day 4 but reversed on day 16, where levels in MB06 surpassed those in MB02-PFHM II. Regarding the phosphorylated form of the AMP-activated protein kinase (p-AMPK), an energy sensor able to suppress the mTORC1 pathway activity under conditions of low energy availability, the levels were double in MB02-PFHM II compared to MB06 at both days (Figures 5A,B).

### Anti-huCD20(hγ1)-IL2no-alpha IC obtained in SFM retains its binding to human CD20 and its cytokine functionality

**FIGURE 6:**
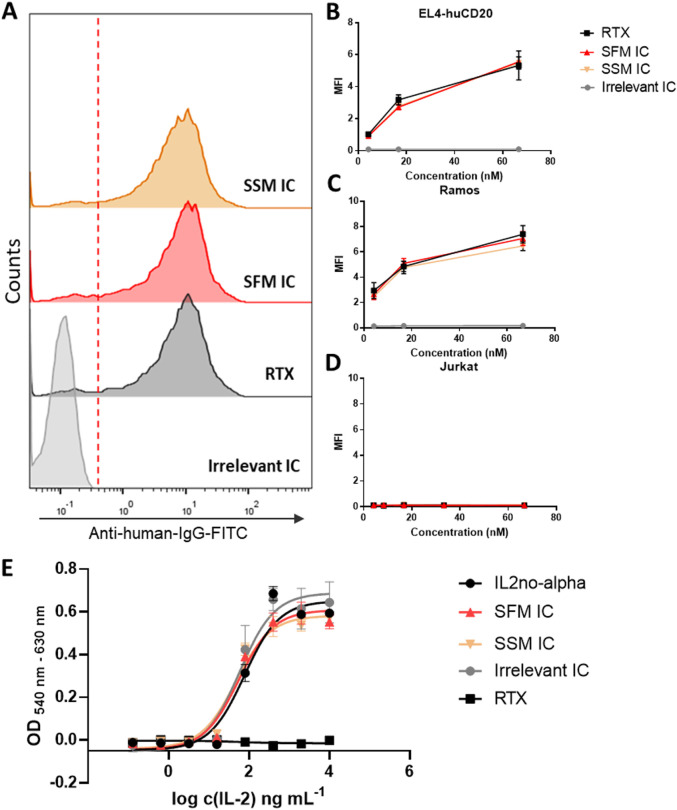
Specific CD20 binding and cytokine-dependent functional activity of anti-huCD20(hγ1)-IL2no-alpha ICs. Flow cytometry staining was performed on EL4-huCD20 and Ramos cells using equimolar concentrations of RTX, SFM, or SSM anti-huCD20(hγ1)-IL2no-alpha ICs or an irrelevant control IC. **(A)** CD20-binding histograms on EL4-huCD20 cells at 66.6 nM and **(B, C)** CD20 recognition curves on EL4-huCD20 and Ramos cells (66 nM–4.12 nM). **(D)** Jurkat leukemia cells were used as control of non-CD20-expressing cells. **(E)** Cytokine-dependent activity of anti-huCD20(hγ1)-IL2no-alpha IC. CTLL-2 cells were cultured in the presence of IL2no-alpha mutein, with RTX, SFM, SSM, or the irrelevant IC. After 48 h, cell proliferation was assessed by alamarBlue and expressed as absorbance values (OD 540–630 nm). Values represent mean ± SEM of cell culture triplicates. At least two independent experiments were performed.

Binding of SFM IC to CD20 was confirmed by flow cytometry on EL4-huCD20 and Ramos cells. The Ramos cell line displayed a high CD20 expression, while EL4-huCD20 cell expression levels were comparable to those from primary human B-NHL or human lymphoma cell lines. SFM IC, SSM IC, and RTX showed a similar recognition profile in EL4-huCD20 (Figures 6A,B) and Ramos cell lines (Figure 6C). Jurkat cells (CD20^−^) (Figure 6D) and the irrelevant IC served as negative controls. Representative histograms from Ramos and Jurkat cell lines are also shown in Supplementary Figure S7. These observations confirmed that SFM IC preserves specific binding to CD20 compared to previously reported results obtained in SSM.

The IL2no-alpha moiety’s activity was validated by CTLL-2 proliferation assay, showing that cell proliferation induced by SFM IC was concentration-dependent and comparable to that induced by SSM IC, and both to IL2no-alpha (Figure 6E). The presented data focus on clone 3E8, but all SFM clones were tested and showed a similar behavior. Supplementary Table S2 summarizes the values of specific activity obtained for each evaluated molecule.

### SFM anti-huCD20(hγ1)-IL2no-alpha retains the antibody effector functions

**FIGURE 7:**
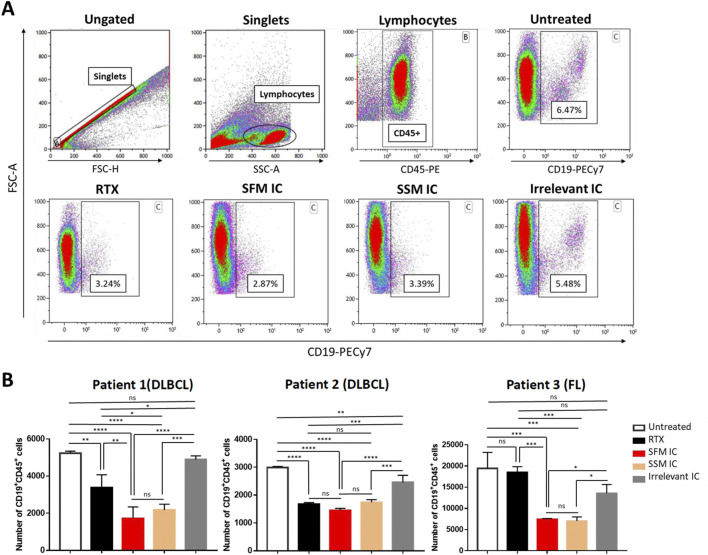
*In vitro* depletion of B cells from B-NHL patients. **(A)** Gating strategy for CD19^+^CD45^+^ cell selection and representative dot plot from B-cell depletion assay performed on patient 2 (DLBCL). It indicates the percentage of CD19^+^CD45^+^ cells from one sample for each treatment. **(B)** Patients’ PBMCs were incubated in presence of 18 nM of RTX, SSM IC, SFM IC, or irrelevant IC for 96 h at 37 °C. The absolute number of B cells within the PBMC was calculated considering that 50,000 cells were analyzed by flow cytometry. Data correspond to three independent samples. Data represent the mean ± SEM (ANOVA, Dunn’s *post hoc* test, *p < 0.05; **p < 0.01; ***p < 0.001; ***p < 0.0001; ns, not significant).

We also investigated whether SFM IC maintained its advantages over RTX in antibody effector mechanisms by flow cytometry. The gating strategy is shown in Figure 7A. *In vitro*, SFM IC depleted malignant B-cells isolated from PBMCs of r/r B-NHL patients, with an effect similar to SSM IC, used as positive control and outperforming RTX in two of three patients (Figure 7B). The irrelevant control showed no effect except in one patient, likely due to direct NK cell activation and unspecific killing through the IL2no-alpha moiety.

### Antitumor activity of the SFM anti-huCD20(hγ1)-IL2no-alpha treatment

**FIGURE 8:**
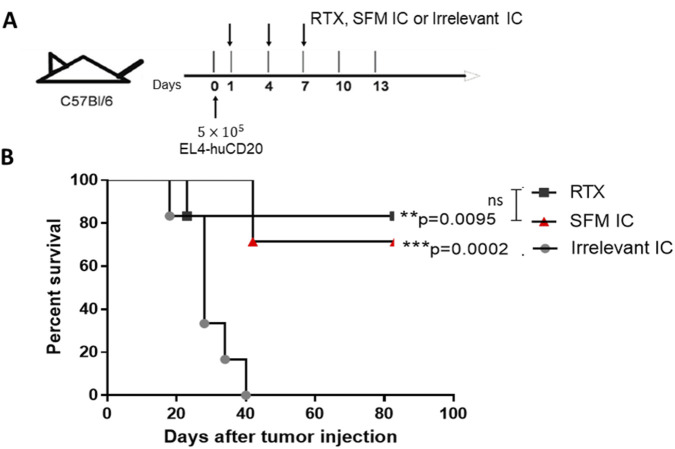
Antitumor effect of SFM anti-huCD20(hγ1)-IL2no-alpha IC on EL4-huCD20 tumor-bearing C57Bl/6 mice. **(A)** Schematic representation of the treatment schedule followed. **(B)** Survival curves of mice with the different treatments as indicated: 150 μg of RTX, 20 μg of SFM IC, or irrelevant IC. Data correspond to a representative experiment of two independent experiments (n = 7 per group) (log-rank test; **p < 0.01, RTX vs. irrelevant IC; ***p < 0.001, SFM IC vs. irrelevant IC; ns, not significant).

The antitumor activity of the anti-huCD20(hγ1)-IL2no-alpha IC obtained in SSM was previously reported in the EL4-hCD20 model (Casadesus et al., 2022). Thus, we evaluated the *in vivo* efficacy of the SFM IC in the same experimental setting. Immunocompetent C57Bl/6 mice were IV injected with 5 × 10^5^ EL4-huCD20 cells on day 0, followed by IP injection on days 1, 4, and 7 of SFM IC (20 μg/injection), irrelevant IC, or RTX (150 μg/injection) (Figure 8A). The RTX dose corresponds to the standard clinical dose of 375 mg m^-2^ (Di Gaetano et al., 2003). Survival rates were similar between RTX and SFM IC-treated mice, replicating the result obtained for the SSM IC (Figure 8B). The isotype control exhibited no biological activity, confirming that the observed effects are specific to the SFM-produced IC and not an artifact of the purification process or residual impurities.

## Discussion

### Development and productivity assessment of SF-adapted CHO-K1 clones

The primary objective of this study was to develop stable CHO-K1 cell lines adapted to serum-free suspension growth that could produce anti-huCD20(hγ1)-IL2no-alpha with good quality and biological activity in appropriate quantities for clinical evaluation. Although highly efficient transient transfection methods have been introduced, the generation of stable mammalian cell lines continues to be essential for the in-depth exploration of mAb and antibody-based molecules (Gupta et al., 2021; Toporova et al., 2023). Furthermore, protein production utilizing stable cell lines can be readily scaled up to achieve elevated product yields (Hunter et al., 2019).

In this study, the recombinant CHO-K1 cells were generated using lentiviral vectors (Godecke et al., 2018; Rives et al., 2024). For the isolation and screening of stable CHO-K1 clones, we followed a methodology based on limiting dilutions, Blasticidin selection, productivity evaluation in T-flask format, and intracellular HC content measurement (Agrawal et al., 2012; Hacker and Balasubramanian, 2016; Lao-Gonzalez et al., 2021). Through various cycles, we successfully obtained three CHO-K1 clones for producing anti-huCD20(hγ1)-IL2no-alpha.

Lentiviral vectors are widely recognized for the rapid generation of stable cell lines due to their ability to transduce a broad range of cell types, achieve multiple integrations into active transcription sites, and enable precise control over copy number via the multiplicity of infection (MOI) (Oberbek et al., 2011; Godecke et al., 2018). Consistently, we observed similar titers among the isolated oligoclones with only small variations, in contrast to those usually obtained by conventional random integration methods, suggesting efficient genomic integration. The system employed in this work shares the well-established safety profile reported for third-generation lentiviral systems (Schambach et al., 2013; Godecke et al., 2018), which split viral genes across several plasmids to prevent recombination and the generation of replication-competent particles (Zufferey et al., 1998). Additionally, the transfer vector is self-inactivating due to a deletion in the 3′ long terminal repeat (LTR), further enhancing biosafety (Miyoshi et al., 1998; Zufferey et al., 1998). During downstream processing, dedicated viral clearance steps, including filtration and chromatography, are implemented to effectively remove residual vector particles, host cell DNA, and other process-related impurities (Strauss et al., 2010; Caballero et al., 2014; Zhang et al., 2014), in accordance with current biopharmaceutical manufacturing practices. The Center for Molecular Immunology has established experience with a similar lentivirus-based platform at production scale, used to obtain the antigen for the SOBERANA 01, SOBERANA 02, and SOBERANA Plus vaccines (Boggiano-Ayo et al., 2023; Toledo-Romani et al., 2023) which have been approved for human use in several countries.

The successful adaptation of all selected clones to suspension culture in SFM enabled the development of a production-relevant culture mode. This transition is particularly advantageous for large-scale bioprocessing, as suspension cultures reduce shear stress and support higher viable cell densities compared to adherent systems (Jang et al., 2022; Mohseni et al., 2023).

### Nutrient-rich medium enhances productivity via cell-cycle modulation and sustained mTORC1 activation in pseudo-perfusion culture

A key challenge in ensuring efficient and scalable cell cultures for industrial use is selecting an optimal culture medium to enhance cell growth and product yield while maintaining quality and cost-effectiveness (Li et al., 2021; Zhang et al., 2024). The 3E8 clone was studied in batch culture for 7 days in both MB02-PFHM and MB06 media, and despite the slightly lower **μ**_max_, it reached a higher maximal VCD in MB06, 1.4-fold higher IVCC, and 1.7-fold higher qp, leading to a 3-fold increase in IC concentration. Under pseudo-perfusion conditions, the results confirmed MB06’s superiority, which influenced key cell culture performance metrics. Cells grown in MB06 medium reached a significantly higher VCD_max_ and IVCC and produced double the product titer compared to those cultured in MB02-PFHM II. This superior performance correlated with a greater μ_max_ and a 1.5-fold increased qp while operating at nearly half CSPR, indicating a more efficient nutrient utilization and process intensification (Walther et al., 2025). Remarkably, the cells used in these studies maintained expression levels similar to those achieved in the T-flask format, demonstrating the stability of IC expression throughout the adaptation process and both culture conditions as well as the predictive value of the T-flask format. Compared to the batch culture, pseudo-perfusion also highlighted the significant advantages of perfusion, including higher VCD and improved qp—critical for process intensification (Pollock et al., 2013) while maintaining the purity and integrity of the product. These benefits arise from the continuous replenishment of nutrients and removal of inhibitory metabolites, enabling longer productive phases and more consistent product quality (MacDonald et al., 2022).

Consistent with these findings, the 3E8 clone in pseudo-perfusion culture also showed a higher percentage of CHO cells in the G_0_/G_1_ phase within the enriched MB06 culture medium compared to MB02-PFHM II during the analyzed days. This G_0_/G_1_ accumulation aligns with studies establishing its positive correlation with enhanced productivity (Avello et al., 2022; Jarusintanakorn et al., 2024). In this phase, cells maintain enhanced biosynthetic and secretory capacity without the pressure of active cell division, contrasting with the resource demands of the S and G_2_ phases (Shahabi et al., 2023). Higher levels of total mTOR and p-mTOR were also found in MB06 during both exponential and stationary phases, along with lower levels of p-AMPK. These findings align with previous studies that demonstrated a direct relationship between mTOR activity and the productivity of CHO cells (Edros et al., 2014; Shahabi et al., 2023) and confirm sufficient energy availability in MB06, enabling the prioritization of anabolic growth (Jaiswal and Kimmel, 2019; Smiles et al., 2024).

The interplay between mTORC1 and AMPK is crucial for maintaining cellular homeostasis. Nutrient-rich environments such as MB06 provide essential amino acids, glucose, and other growth factors that activate signals to recruit mTORC1 complex to lysosomal membranes. Under nutrient-rich conditions, AMPK is downregulated to prevent catabolic pathways from interfering with growth (Jaiswal and Kimmel, 2019; Audet-Walsh et al., 2020). Additionally, the continuous nutrient supply, essential for cell growth and protein production, drives persistent anabolic signaling, even in the high-density stationary phase (Edros et al., 2014; Kolaczkowsk et al., 2024) and contrasting to the decline observed in other culture formats like batch or fed-batch (McVey et al., 2016). This consistently strong anabolism explains the concurrent IVCC and productivity gains observed in the MB06 perfusion system with a lower CSPR, reflecting an optimal balance between growth and protein synthesis under favorable nutritional conditions. Nevertheless, further research is required into the dynamics of this signaling pathway throughout the entire culture period.

### SF production preserves the structural integrity and biological function of the IC

Maintaining protein integrity remains another primary concern in recombinant protein production, as culture medium composition impacts protein stability during both production and purification. Recent studies demonstrate that specific amino acid concentrations and certain metabolites influence immunoglobulin folding and aggregation (Kumar et al., 2024; Lee et al., 2025).

Analysis of SFM IC via SDS-PAGE and Western blot have revealed no significant differences in structural integrity or molecular weight profiles between SF and FBS-supplemented conditions. Notably, SEC-HPLC studies showed substantially reduced protein aggregation in SFM after a single step of protein A purification, with monomeric fractions increasing from 70%–80% to >90%. DLS further confirmed a monodisperse population (PDI <0.1) according to established criteria, with a hydrodynamic diameter consistent with the expected size of 180 kDa (Stetefeld et al., 2016). This represents a critical quality improvement, as high aggregation compromises therapeutic efficacy through immunogenicity and reduced bioactivity (Pham and Meng, 2020; Lundahl et al., 2021). The mechanism behind this reduction in aggregation may be multifactorial. Proteins, hormones, and growth factors contained in FBS can influence cell metabolism and protein synthesis pathways, potentially leading to aggregation (Lee et al., 2022); in contrast, SFM is often designed to provide a more controlled and optimized environment, favoring protein quality, folding, and assembly (Li et al., 2021; Lee et al., 2025).

Having established the improved profile of SFM IC, its biological potency was subsequently evaluated. Although transitioning to SFM could theoretically impact IC’s biological potency by altering critical attributes like folding or post-translational modifications, functional assays revealed no significant differences in target recognition or CTLL-2 proliferation. However, such modifications could directly affect B-cell depletion mechanisms by modulating effector cell engagement or the capacity to expand and activate NK and CD8^+^ T cells in the tumor microenvironment, ultimately impacting therapeutic efficacy (Trzos et al., 2023; Hao et al., 2025).

A B-cell depletion assay with PBMC samples from r/r B-NHL patients was performed, as it integrates all described mechanisms of action of CD20 antibodies such as direct cell killing, antibody-dependent cell cytotoxicity, and antibody-dependent cell phagocytosis (Ysebaert et al., 2015). In this scenario, SFM IC showed similar activity to SSM IC and was superior to RTX in two cases. The maintained efficacy was also confirmed in an immunocompetent mouse model. Consequently, the consistent biological profile confirms that the SF process successfully preserves these complex attributes, ensuring a therapeutically competent molecule, in line with literature on SF systems (Jukic et al., 2016; Li et al., 2021).

In conclusion, we obtained IC-producing CHO-K1 clones adapted to SF conditions, with a productivity that provides a realistic starting point for scale-up considering doses used in clinical trials for similar therapeutics (Raeber et al., 2023) while maintaining its essential physicochemical and biological properties. Nevertheless, large-scale manufacturing requires critical consideration of time, cost, and production efficiency, and understanding how culture media composition affects signaling pathways can guide strategies such as mTOR signaling engineering or culture media supplementation, impacting directly in biotechnological outcomes. Taken together, these data align with current trends in biomanufacturing where nutrient-rich and animal component-free media formulations are leveraged to maximize both cell density and product yields, enhancing the consistency and reliability of therapeutic protein manufacturing.

## Data Availability

The original contributions presented in the study are included in the article/Supplementary Material; further inquiries can be directed to the corresponding author.
