# Supplementary material for: Serum-free production of anti-huCD20(hγ1)-IL2no-alpha immunocytokine: a promising therapeutic for B-NHL
Source: Front Bioeng Biotechnol. 2026 Apr 15;14:1773646. doi: 10.3389/fbioe.2026.1773646 (PMC13126307; doi:10.3389/fbioe.2026.1773646)
Supplement: Supplementary file 1 [file DataSheet1.pdf]

Supplementary Material

Supplementary Figures

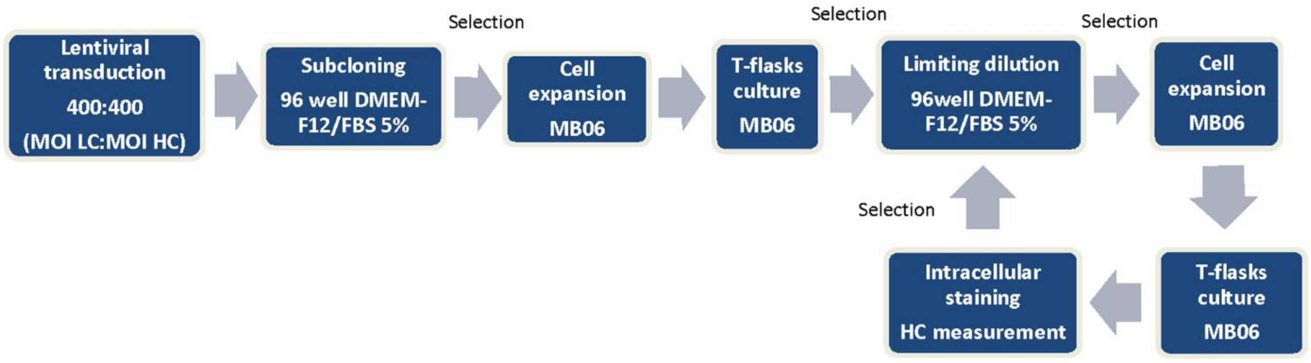

Supplementary Figure 1 Strategy for generating CHO-K1 clones producing anti-huCD20(hg1)-IL2no-alpha IC

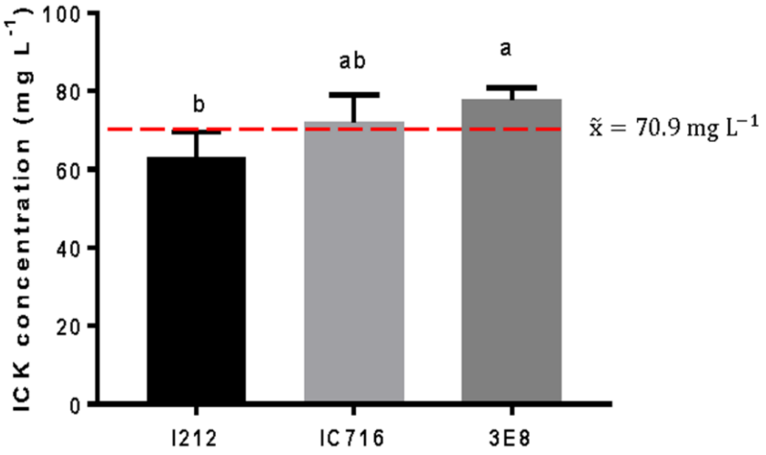

Supplementary Figure 2 Anti-huCD20(hg1)-IL2no-alpha IC production in 100 mL batch cultures of three selected CHO-K1 clones cultured in MB06 medium. Batches were performed in biological triplicates, and collected at 7th day.

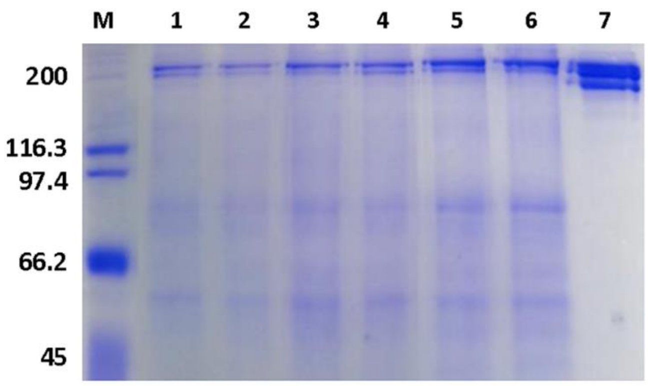

Supplementary Figure 3 SDS-PAGE analysis of CHO-K1 clone 3E8 cultured in pseudo-perfusion conditions on days 4, 6, and 16 under non-reducing conditions. (Lane 1-3: supernatants from days 4, 6, 16 in MB02-PFHM II medium; lane 4-6: supernatants from days 4, 6, 16 in MB06 medium; lane 7: IC purified from 3E8 pseudo-perfusion; M: Molecular Weight Marker). The gel was cropped to remove irrelevant lanes; brightness and contrast were adjusted uniformly across the entire image.

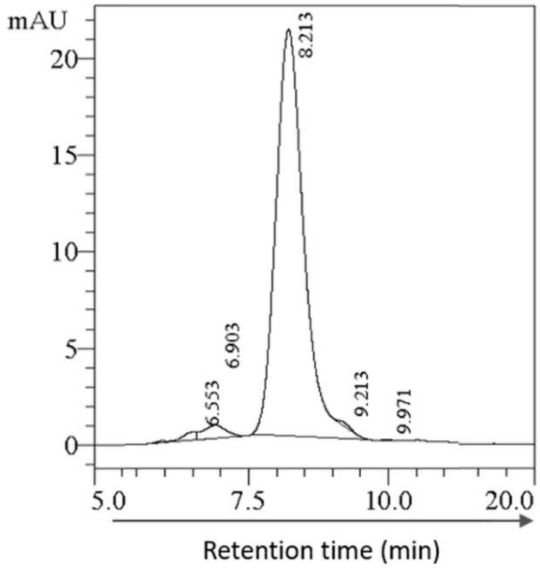

Supplementary Figure 4 SEC-HPLC of the anti-huCD20(hg1)-IL2no-alpha IC purified from the CHO-K1 clone 3E8 pseudo-perfusion.

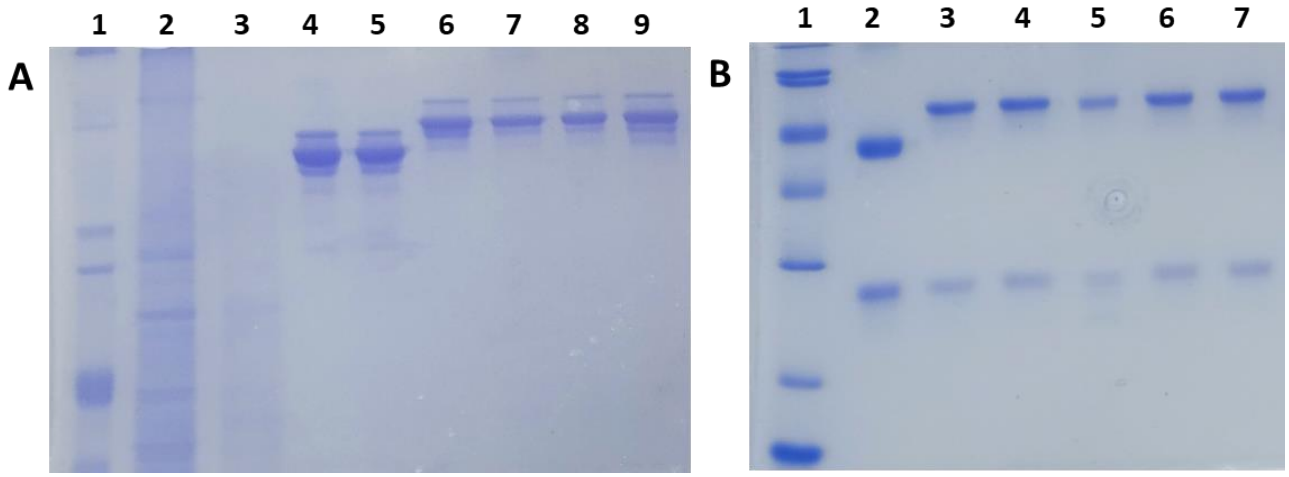

Supplementary Figure 5 Entire original SDS-PAGE gels of purified ICs (from **Figure 2)** under non-reducing (**A**) and reducing conditions (**B**). Panel A; Lane 1: Broad Range molecular weight marker, 2-3: supernatants from other proteins, 4-5: RTX (two lots), 6: SFM IC I212, 7: SFM IC I216, 8: SFM IC 3E8, 9: SSM IC. Panel B; Lane 1: Broad Range molecular weight marker, 2: RTX, 3: SFM IC I212, 4: SFM IC I216, 5: SFM IC purified from an oligoclone, 6: SFM IC 3E8, 7: SSM IC

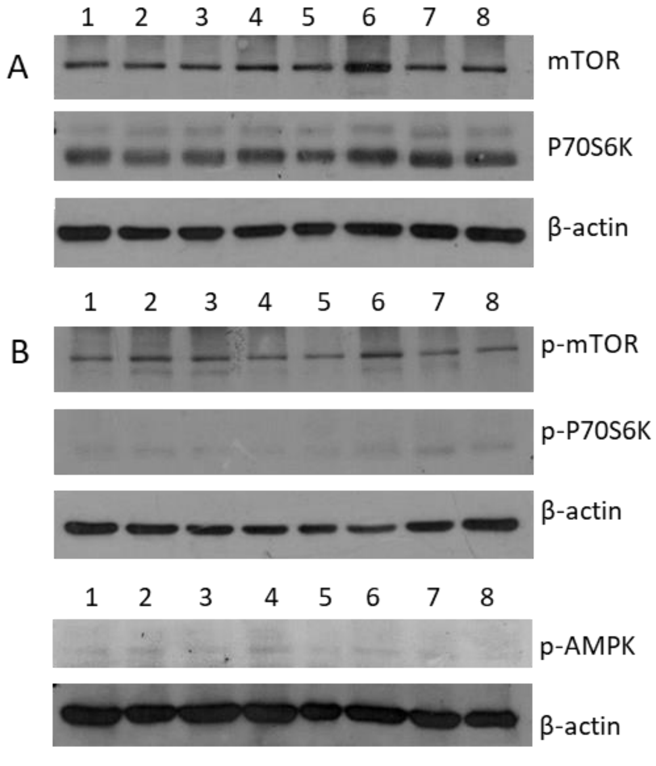

Supplementary Figure 6 Entire original Western blots from **Figure 5** total (**A**) and phosphorylated proteins (**B**). (mTOR, P70S6K and β-actin Lane 1: MB02-PFHM II Day 4 (perfusion 1), Lane 2 MB02-PFHM II Day 4 (perfusion 2), Lane 3 MB02-PFHM II Day 16 (perfusion 1), Lane 4 MB02-PFHM II Day 16 (perfusion 2), Lane 5: MB06 Day 4 (perfusion 1), Lane 6 MB06 Day 6 (perfusion 1), Lane 7 MB06 Day 6 (perfusion 2) and Lane 8 MB06 Day 16 (perfusion 1). p-mTOR, p-P70S6K and β-actin Lane 1: MB02-PFHM II Day 4 (perfusion 1), Lane 2 MB02-PFHM II Day 4 (perfusion 2), Lane 3 MB06 Day 16 (perfusion 1), Lane 4 MB02-PFHM II Day 16 (perfusion 1), Lane 5: MB06 Day 4

(perfusion 1), Lane 6 MB02-PFHM II Day 6 (perfusion 1), Lane 7 MB06 Day 6 (perfusion 1) and Lane 8 MB06 Day 16 (perfusion 2). p-AMPK Lane 1: MB02-PFHM II Day 4 (perfusion 1), Lane 2 MB02-PFHM II Day 4 (perfusion 2); Lane 3 MB02-PFHM II Day 16 (perfusion 1), Lane 4 MB02-PFHM II Day 16 (perfusion 2), Lane 5: MB06 Day 4 (perfusion 1), Lane 6 MB02-PFHM II Day 6 (perfusion 1), Lane 7 MB06 Day 16 (perfusion 1) and Lane 8 MB06 Day 16 (perfusion 2)).

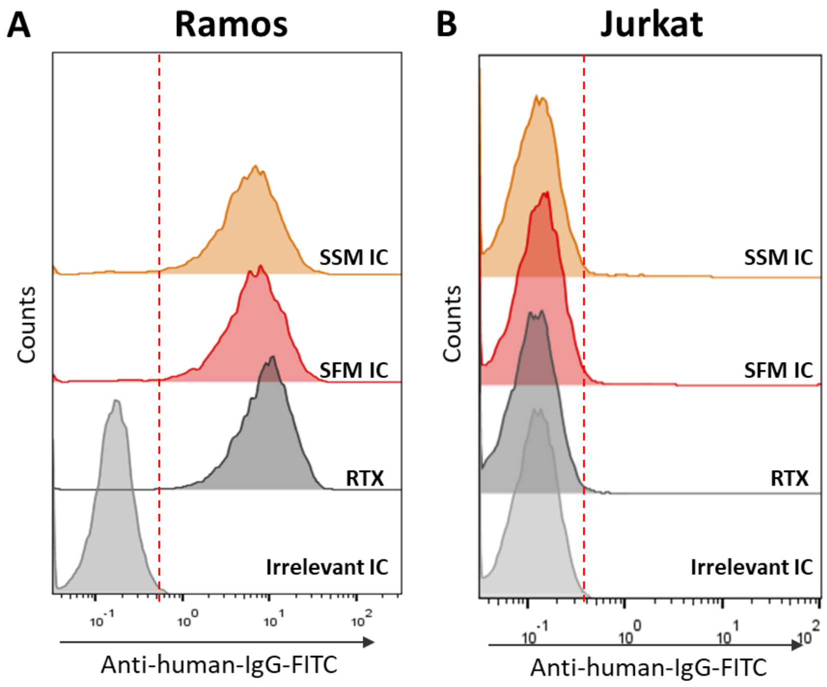

Supplementary Figure 7 CD20 recognition histograms on Ramos (A) and Jurkat (B) cells at 66.6 nM.

Supplementary Tables

Supplementary Table 1 Retention times and monomer percentages of three 100 mL batches from each clone

Clone	Batch	Retention time (min)	Monomeric fraction (%)
**I212**	1	7.785	94.53
	2	7.796	98.14
	3	7.741	92.46
**IC716**	1	7.777	94.97
	2	7.782	95.14
	3	7.861	95.45
**3E8**	1	7.792	94.17
	2	7.795	96.10
	3	7.797	96.17
**Thyroglobulin**		6.146	68.11
**Aldolase**		8.480	70.03

Supplementary Table 2 Average cumulative particle diameter and polydispersity index (PDI) of SFM IC

Run	Cummulative diameter (nm)	PDI
**1**	15.3	0.029
**2**	16.7	0.010
**3**	16.2	0.015

Supplementary Table 3 Specific IL-2 activity obtained for each molecule evaluated in CTLL-2 assay

	R^2	Specific Activity [UI]
**IL-2no-alpha**	0.950	1.30×10^4
**SFM IC**	0.968	1.78×10^4
**SSM IC**	0.966	2.15×10^4
**Irrelevant IC**	0.943	1.72×10^4
